# Tiling Array Analysis of UV Treated *Escherichia coli* Predicts Novel Differentially Expressed Small Peptides

**DOI:** 10.1371/journal.pone.0015356

**Published:** 2010-12-23

**Authors:** Gard O. S. Thomassen, Ragnhild Weel-Sneve, Alexander D. Rowe, James A. Booth, Jessica M. Lindvall, Karin Lagesen, Knut I. Kristiansen, Magnar Bjørås, Torbjørn Rognes

**Affiliations:** 1 Centre for Molecular Biology and Neuroscience (CMBN) and Department of Microbiology, Rikshospitalet, Oslo University Hospital, Oslo, Norway; 2 Centre for Molecular Biology and Neuroscience (CMBN) and Department of Microbiology, University of Oslo, Oslo, Norway; 3 Department of Informatics, University of Oslo, Oslo, Norway; 4 Institute of Clinical Biochemistry, University of Oslo, Oslo, Norway; University of Massachusetts Medical School, United States of America

## Abstract

**Background:**

Despite comprehensive investigation, the *Escherichia coli* SOS response system is not yet fully understood. We have applied custom designed whole genome tiling arrays to measure UV invoked transcriptional changes in *E. coli*. This study provides a more complete insight into the transcriptome and the UV irradiation response of this microorganism.

**Results:**

We detected a number of novel differentially expressed transcripts in addition to the expected SOS response genes (such as *sulA*, *recN*, *uvrA*, *lexA*, *umuC* and *umuD*) in the UV treated cells. Several of the differentially expressed transcripts might play important roles in regulation of the cellular response to UV damage. We have predicted 23 novel small peptides from our set of detected non-gene transcripts. Further, three of the predicted peptides were cloned into protein expression vectors to test the biological activity. All three constructs expressed the predicted peptides, in which two of them were highly toxic to the cell. Additionally, a remarkably high overlap with previously *in-silico* predicted non-coding RNAs (ncRNAs) was detected. Generally we detected a far higher transcriptional activity than the annotation suggests, and these findings correspond with previous transcription mappings from *E. coli* and other organisms.

**Conclusions:**

Here we demonstrate that the *E. coli* transcriptome consists of far more transcripts than the present annotation suggests, of which many transcripts seem important to the bacterial stress response. Sequence alignment of promoter regions suggest novel regulatory consensus sequences for some of the upregulated genes. Finally, several of the novel transcripts identified in this study encode putative small peptides, which are biologically active.

## Introduction

UV irradiation is one of the most common treatments used to study cellular responses to DNA damage. In *Escherichia coli* UV irradiation transiently blocks DNA replication and leads to the induction of a physiological response termed the SOS response [1]. More than 40 genes induced by the SOS response are negatively regulated by the LexA repressor which binds to operator sequences upstream of genes or operons [2]. After UV irradiation RecA protein binds to ssDNA formed by a replication block, leading to the generation of a RecA-ssDNA nucleoprotein filament that is able to mediate auto-proteolysis of the LexA repressor. As a consequence, a decline in LexA concentration occurs, and SOS regulated genes are induced [1]. In addition to the LexA repressed genes, a large number of genes are known to have a LexA independent change in gene expression following UV irradiation [3]. In that study, *E. coli* microarrays, carrying 95.5% of all annotated open reading frames, were utilized to identify changes in gene expression after UV irradiation [3].

During the past decade, whole-genome studies have proven that microarrays are a successful means to study the expression of entire genomes, which has enabled the investigation of global gene expression patterns in organisms such as yeast, human, mouse and bacteria [4]–[8]. Many recent tiling array transcriptome studies report a level of transcription far exceeding their respective annotations [9], [10]. The question of whether these transcripts represent genes, novel mRNAs, ncRNAs, or other important RNA products still remains largely unknown.

In this paper, a wide range of novel short *E. coli* transcripts is presented, of which many are differentially expressed in response to UV exposure. We also present all the non-differentially expressed transcripts. The array data have been analysed both according to the present annotation and independently of any previous annotation, giving a more unbiased approach of analysis, and all transcripts have subsequently been categorized. Additionally we present a plain transcriptome mapping of the reference and the treated bacteria. A promoter study of all upstream regions of detected transcripts overlapping known genes is also presented. Finally, all differentially and similarly expressed transcripts without any overlap to annotated genes were screened for possible open reading frames. Through the experimental identification of all RNAs expressed or suppressed under the influence of UV irradiation, a more thorough understanding of the regulatory networks is gained, shedding further light on the full *E. coli* transcriptome and its role as an important model organism.

## Materials and Methods

### Strain and growth conditions

The experiments were carried out in strains MG1655 (*LAM*, *rph*) [11]–[13], AB1157 (*arg*, *his*, *leu*, *pro*, *thr*, *ara*, *gal*, *lac*, *mtl*, *xyl*, *thi*, *tsx*, *rpsL, supE* and *kdgK*) [14], ER2566 (lacZ::T7 gene1) (New England Biolabs, Ipswich, MA, USA), DM49 (*lexA3*) (LexA ind-) derived from AB1157 [15] and GW2730 (*lexA71::Tn5*) (LexA(Def)) [16].

Expression plasmids pET28b-3xFLAG-D1, -D2 and -D3 contains the D1, D2 and D3 open reading frames inserted in the NcoI–HindIII restriction sites of the pET28b(+) vector (Novagen, Madison, WI, USA). The plasmids were purchased from GenScript Corp. (Piscataway, NJ, USA).

*Escherichia coli* K-12 MG1655 from overnight cultures were diluted 1∶500 in K-medium (1xM9, 1.2% glucose, 1.25% casamino acids (dCAA), 1 mM MgSO_4_, 0.1 mM CaCl_2_) and subsequently grown at 37°C. Cells were grown in 100 ml batch cultures in 500 ml Erlenmeyer flasks with aeration by rotary shaking. At OD_600_ = 0.5 the SOS response was induced in 50 ml of the culture by UV irradiation (50 J/m^2^). The reference culture was treated similarly but unexposed. Cells were harvested by centrifugation 15 minutes after UV exposure.

### RNA isolation, cDNA synthesis and target labeling

RNA was isolated as described by [17]. In short, total RNA was extracted from the cells using a procedure based on the Trizol reagent combined with RNeasy columns (QIAGEN). 1 ml of Trizol was added per 50 ml cell culture and incubated at room temperature for 5 minutes. 0.2 ml chloroform was added per ml of Trizol and the sample was mixed before centrifugation at 12 000× g and 4°C. The aqueous phase was slowly added 1∶1 to 70% EtOH to avoid precipitation. The sample was further loaded to the RNeasy column, washed and DNase treated according to the RNeasy protocol (QIAGEN). Isolated RNA was resuspended in RNase free water and quantitated using NanoDrop® ND-1000 UV-Vis Spectrophotometer. The RNA was further converted into fragmented and labelled cDNA according to the Affymetrix ‘Prokaryotic Sample and Array Processing protocol version 701029 Rev. 4. Briefly, cDNA was prepared from 20 µg total RNA via reverse transcription with random hexamers. 3.5 µg of the resulting cDNA was fragmented using DNaseI and terminal labelled with Biotin, 2.94 µg were hybridized to arrays overnight at 45°C. Five biological replicates were hybridised for each of the two conditions, hybridisation was performed as per the manufacturers’ instructions.

During the initial analysis of the data we discovered widespread antisense regulation in agreement with previous tiling studies [9], [18], [19]. Further investigations using Northern blotting (data not shown) suggested that these transcripts are mostly artefacts. As shown by Perocchi *et al.* in 2007 [20]; widespread antisense transcription may be detected due to second strand cDNA synthesis during reverse transcription in the sample preparation procedure if actinomycin D is not used. Based on Perocchi *et al.'*s findings from *Saccharomyces cerevisiae* and our Northern blotting experiments, we concluded that the majority of the detected antisense transcripts were artefacts. Exceptions might be transcripts that are non-correlated to the sense strand, and where the sense strand is poorly annotated, i.e. in cases of annotation on the wrong strand. Due to the uncertainty of the detected antisense transcripts they were removed from this report.

### Reverse transcriptase real-time quantitative polymerase chain reaction

cDNA was generated from the DNaseI treated total RNA solutions using a volume equivalent to 1 µg and using the High Capacity cDNA Reverse Transcription Kit (ABI). The reactions were carried out according to the manufacturer's instructions. The Power SYBR® Green PCR MasterMix was used in conjunction with the StepOnePlus™ Real-time PCR System (ABI) and the equivalent of 5 ng of the cDNA to generate the real-time plots which were then processed by its associated software, StepOne™ Software v2.0.1, in order to generate the cycle threshold (*C_t_*) values. All steps were carried out as per the manufacturer's instructions. All qPCR primers for the candidate transcripts were designed using Primer Express® 3.0 (ABI), the majority of which were automatically generated, the remaining were designed manually using the same software. All samples were run in quadruplicates and in addition four independent parallels were used to generate the data for each transcript of interest. The *C_t_* value of the transcript of interest was subtracted from that of *rrsB* (16S ribosomal RNA), a stably expressed gene, in order to give a value specific to the transcript. The value generated from a sample treated with UV was subtracted from that generated from reference parallel order to observe any modulation. The visual representation of the transcript specific values showing fold change, assumes a PCR efficiency of two. The specificity of the PCR reactions was determined from dissociation curves generated after the qPCR reactions. Controls included no template controls (NTC) and no RT controls.

### Array design, normalization and scaling

The design, normalisation and analysis methods used on the applied arrays are thoroughly described in Thomassen *et al.*2009 [21]. In short, the design focuses on uniform binding affinities on all probes and a very high coverage of intergenic regions (down to 7 nt resolution in intergenic regions and 19 probes pr coding region, similar coverage on each strand). The normalisation method is based on a minimized normalisation (as suggested by Royce *et al.* [22]) focusing on removing significant outliers from the data, the data was subsequently baseline shifted. The minimum signal intensity of a true probe signal was set to 9.0 on a log_2_ scale and therefore all measured intensities below 9.0 are considered uncertain as these probe values are inseparable from background noise, and the minimum distances of a differentially and a similarly expressed transcript was set to 25 and 36 respectively (for details see Thomassen *et al*. [21]). The microarray data and the array definition have been submitted to Gene Expression Omnibus [23] with accession number GSE 13829 (data) and GPL 7714 (array).

### Annotation used in data analysis

The *E. coli* K12 MG1655 (NC_000913) genome annotation used in the post-processing of the data documents a total of 4306 annotated genes (including tRNAs, rRNAs and ncRNAs) in the *E. coli* genome, of which 4263 genes (including all the 63 ncRNAs) were probed by one or more probes. All expressed transcripts detected were sorted into one of the following groups: 1. Genes (one or more nucleotides overlap with an annotated gene), 2. Possible 5′UTRs (≤100 nt upstream of annotated gene), 3. Possible 3′UTRs (≤100 nt downstream of annotated gene), 4. Possible operon elements (fulfils number 2 and 3), and 5. Novel transcripts (≥100 nt from annotated gene upstream and downstream). (See Thomassen *et al.*, [21] for details about the annotation and the classification of transcripts).

#### Array data analysis

Three different analysis methods were applied in order to get full comprehension of the data. The first method is an annotation based method that immediately reveals whether the results are reasonable by comparing annotated genes only. This approach uses a t-test to compute the probability of differential expression between annotated genes by comparing the probe intensity values for all probes targeting the gene in the reference and the treated sample. As a positive control, this annotation based approach should reveal known SOS response genes as *sulA* and *recN* to show strong upregulation, while known housekeeping genes (*gapA*, *rrsB*) should remain fairly similarly expressed. The second method is a sliding window based, a novel annotation independent method, which segments the data and performs a reference versus treated bacteria comparison (referred to as the sliding window method). The selection of differentially and similarly expressed regions is based on t-tests computed with multiple window sizes. Finally, the third method detects absent and present calls (i.e. plain transcriptome mapping) with no reference versus treated comparison. All of the above analysis methods are described in Thomassen *et al.* 2009 [21]. The thresholds for differential expression require a probability for differential expression ≥0.95 combined with a log_2_ fold change of at least 0.5. All remaining transcripts expressed above background level in both conditions are considered similarly expressed. The phrase “to touch” a gene in this analysis is used when a transcribed region overlaps an annotated gene with one or more nucleotides. Additionally, the nature of the annotation independent analysis method results in that parts of an annotated gene might be reported as partly differentially expressed, partly similarly expressed and/or undetected.

#### Search for open reading frames

All differentially and similarly expressed transcripts detected in this study that do not overlap any part of an annotated protein coding transcript, were used as the basis for this search. All the nucleotide sequences were submitted to GeneMark.hmm for prokaryotes (v.2.4) [24] to detect open reading frames. GeneMark.hmm is a hidden Markov model (HMM) based gene finder, which searches for ORFs by investigating how well a nucleotide sequence fits into the HMM. The HMM is trained on the nucleotide sequences of prokaryotic genes, thereby the algorithm also accounts for codon usage. To refine the quality of the ORF start site prediction, the algorithm subsequently searches the −4 to −19 region of all possible start codons for a possible ribosome binding site (RBS). The RBS search is based on a multiple sequence alignment of genes from *E. coli* with a documented RBS. The Jpred software [25] was applied for secondary structure predictions of the ORFs, and protein BLAST was used for amino acid sequence homology searches. Additionally TMHMM was used for prediction of possible transmembrane protein structures [26], [27].

## Results

### Regulation of annotated transcripts

The annotation guided method (described in the “Data analysis” section) indicates that 632 known genes are differentially expressed between untreated and UV exposed samples, of which 170 are upregulated after UV induction. Many of most prominent genes are, as expected, known SOS genes (*umuD*, *recN*, *umuC*, *sulA*, *tisB*, *dinI*). These numbers indicate a general downregulation of the entire transcriptome, combined with a focused induction of a specific set of damage response genes, *lexA* dependent and others. Eleven of the 632 differentially expressed genes are ncRNA genes, of which seven are upregulated while the remaining four are downregulated. For two of the upregulated ncRNAs, *istR-2* and *rprA*, no expression above the microarray background signal can be detected in the reference transcriptome. The “turn-on” of *istR-2* is in agreement with previous findings [28]. Interestingly, *rprA* is known to increase the translation of *rpoS*, which has, among other functions, the ability to regulate bacterial response to stress conditions [29]–[31]. It is tempting to speculate that these two ncRNA (amongst others) can be direct targets of the SOS response pathway but this remains to be shown. All detected transcripts (using both the annotation based method and the sliding window method) have been classified as defined in the Materials and Methods section, and are available at GEO [23] (accession number GSE13829).

### Regulation of all expressed transcripts

**Figure 1:**
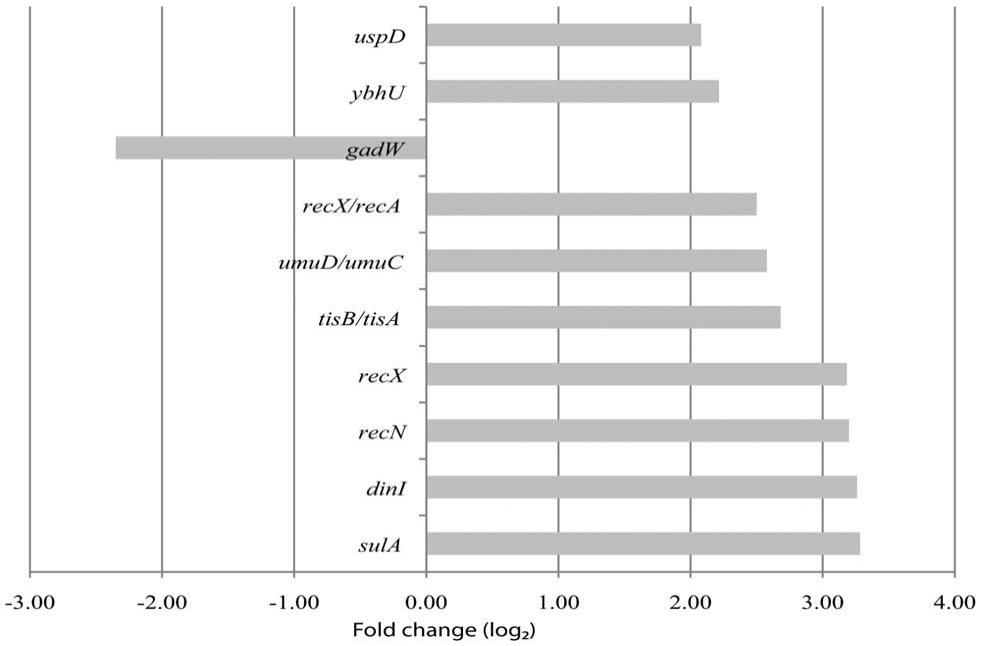
Top ranking differentially expressed annotated transcripts. The 10 most prominent differentially regulated transcripts that partly or completely overlap known genes. These transcripts were detected by the sliding window method. *recX* is listed twice and some genes are listed in pairs (e. g. *umuC/umuD*), this is due to the nature of the annotation independent segmentation algorithm. The probability of differential regulation for these genes is >0.999, and the fold-change is given on a log_2_ scale.

**Figure 2:**
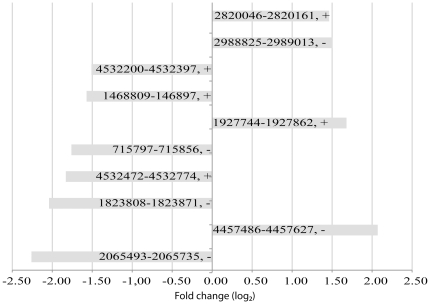
Top ranking differentially expressed novel transcripts. The 10 most prominent differentially regulated novel transcripts; these transcripts are located ≥100 nt from annotated genes in both directions. These transcripts were detected by the sliding window method. The start and end position as well as the orientation on the genome for the transcribed regions are indicated on the bars. The probability of differential regulation for these genes is >0.999, and the fold-change is given on a log_2_ scale.

A total of 2413 transcripts were detected as differentially expressed by the sliding window method (Table 1). The top candidates (Figure 1) of these transcripts are all known genes associated to the SOS response. From the numbers of up- versus downregulated (170 versus 462) transcripts it is easily seen that the bacteria upregulates a selection of stress response genes, while the majority of modulated genes are downregulated to focus on the repair process. Of the remaining 518 up- or downregulated transcripts 227 are classified as possible UTRs or operon elements. From the transcripts overlapping more than one gene additionally 386 possible UTR and operon elements where detected. RegulonDB [32] (a database of *E. coli* operons and other *E. coli* related data) lists 813 operons of which 614 overlap with operons detected in the present study. Finally, 291 differentially expressed transcripts are presented as potential novel transcripts without any suggested function or present annotation, the top candidates are shown in Figure 2. The detected novel differentially and similarly expressed transcripts are evenly distributed along the chromosome, with no obvious hotspots recorded.

**Table 1:** Classification of expressed transcripts.

	Differentially expressed		Similarly expressed	
Classification	Genes *	Transcripts	Genes *	Transcripts
Annotated on same strand	743(14)	1895	4017(52)	4597
Potential operon elements	-	105	-	1381
Potential 5′UTRs	-	303	-	1750
Potential 3′ UTRs	-	252	-	1707
Potential novel transcripts with no predicted function	-	291	-	1909

*Numbers in parenthesis indicate the number of genes annotated as ncRNAs. See Materials and Methods for the classification details. The number for operon elements and UTRs is the sum of non-gene overlapping and parts of gene-overlapping transcripts classified as a UTR or an operon element.

**Figure 3:**
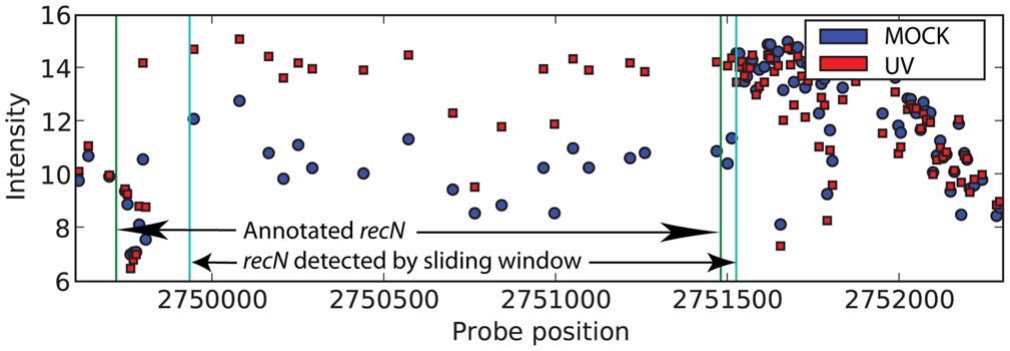
Sense strand transcriptional activity in the *recN* region. Expression measurements surrounding the known SOS response gene *recN*. The sliding window detects the coding part of *recN* with an almost perfect overlap to the annotation, and the upregulation of *recN* in the UV treated *E. coli* is easily seen. Intensity is given on a log_2_ scale.

**Figure 4:**
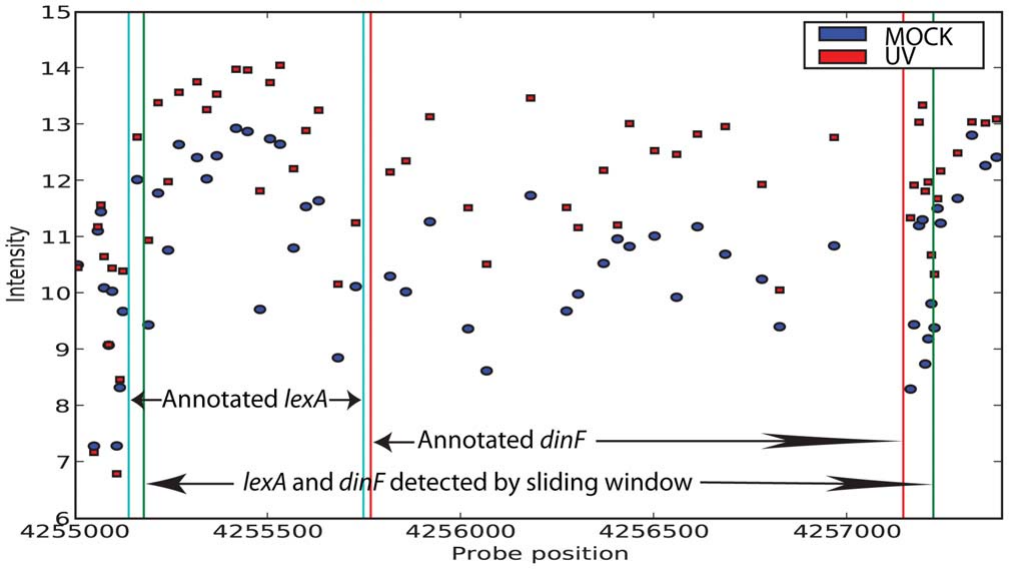
Expression measurements surrounding *lexA* and *dinF*. Intensity levels of all probes for the reference (blue circles) and treated (red squares) transcriptome in the region surrounding the *lexA* and the *dinF* genes. The sliding window method detects these two genes as one continuous differentially transcript demarcated by the green vertical bars. The UV induced upregulation of *lexA* and *dinF* is easily seen from the plot, and the transcript detected starts approximately at the annotated start of *lexA* (leftmost blue vertical bar) and ends slightly downstream of the annotated end of *dinF* (rightmost red vertical bar). Intensity is given on a log_2_ scale.

The analysis quality is strengthened by precise overlaps when comparing annotations of well characterised genes to the findings of the sliding window approach (Figure 3). As the algorithm is annotation independent it may also concatenate closely located transcripts that behave similarly (Figure 4), indicating possible operons or re-annotation of transcript start and stop sites. We therefore believe that the annotation based method *together* with the sliding window approach gives far more insight into the information presently available for the *E. coli* genome and its behaviour in UV stress response. This notion is supported by several recent studies on different organisms that report more transcription than the present annotation suggests [9], [10], [19].

[21] [20] [19] [18]

### Comparison to previous predictions of ncRNAs

At the time of writing there were 63 annotated ncRNAs in RFAM [33]. In addition to the annotated and verified ncRNAs, several studies have suggested various numbers of ncRNAs. In 2003, a list of 1235 *E. coli* ncRNA candidates (including previous predictions) was published by Hershberg *et al.* [34], additionally a list of 306 computationally predicted ncRNAs was published by Saetrom *et al.* in 2005 [17]. A total of 171 of the candidates from the latter study overlap with one or more of the predictions found in the Hershberg list [34]. When comparing our list of transcripts to these two lists of ncRNA candidates, a remarkable overlap is found (Table 2). In the reference dataset we found a total of 254 out of the 306 ncRNAs identified by Saetrom *et al.* [17], and 1131 of the 1235 candidates from the Hershberg compilation [34] are detected, which correspond to a detection level of 83% and 92%, respectively. This indicates that the sliding window transcript detection algorithm has a high sensitivity for ncRNA transcripts. An overview of the overlap to the two previous studies is found in Table 3. Fourteen differentially regulated transcripts detected in this study overlap with previous ncRNA candidates from both Saetrom *et al.* [17] and Hershberg *et al.* [34] (Table 2). Seven of these are detected within transcripts that also span other annotated genes and/or ncRNAs (examples include *dinQ* and *istR-2*, as well as candidate ID R1, R2, R4-R6, R11 and R12 in Table 2), while the rest are located inside transcripts that are located ≥100 nt from any upstream and downstream annotation (Table 2).

**Table 2:** Differentially expressed transcripts overlapping minimum two previous ncRNA predictions.

ID	Saetrom ID	Prev. predictions by	Overlap (%) with Saetrom prediction	Transcript length	Fold-change
R1	I293	[47], [48]	44	53	+2.20
R2	I179	[47]	22	302	−1.83
R3	I227	[36]	100	61	+1.81
R4	I253	[36]	100	197	−1.51
R5	I151	[47], [49]	40	197	−1.51
R6	I186	[48], [49]	32	36	−1.40
R7	I040	[49]	100	1313	−1.26
R8	I238	[49]	66	188	+1.06
R9	I056	[49]	100	504	−1.06
R10	I247	[35], [49]	22	56	+1.01
R11	I152	[35]	70	54	−0.94
R12	I136	[49]	44	49	−0.93
R13	I104	[49]	18	57	−0.84
R14	I104	[49]	38	66	−0.70

14 differentially expressed transcripts (fold-change is log_2_) overlap with predictions of ncRNAs made by Saetrom *et al*. [17] and with previous predictions found in the list compiled by Hershberg *et al.* [34]. The probabilities of differential expression for these transcripts are greater than 99.5%. The transcripts were detected by the sliding window method. All candidate transcript details can be found by the ID column in supplementary info at GEO (GSE13829).

**Table 3:** Comparison between predicted ncRNAs and detected transcripts.

Publications	Predicted in total	Present in reference cells	Present in treated cells	Similarly expressed	Differentially expressed	Not found in this study
Saetrom *et al.* [17]	306	254	249	224	29	43
Hershberg *et al.* [34]	1235	1131	1124	1070	93	88
Intersection	171	140	141	128	13	24
Union	1370	1245	1232	1166	109	107

Overview of the number of expressed transcripts that have previously been predicted as ncRNAs, counted by unique IDs in the respective publications.

Intersection represents the ncRNAs candidates present in both, while union represents the number of ncRNA candidates present in either of the two studies (Saetrom and Hershberg).

### Comparison to previous microarray studies

As mentioned, a number of tiling studies on other species have revealed measurements of a significantly higher level of transcription than previously suggested. The transcriptional data from this study is in agreement with these findings. To further verify our data, we performed a comparison with a time-point study published by Courcelle *et al.* in 2001 on UV treated versus wild type gene expression of *E. coli* MG1655 (similar to this study) [3]. Their study was conducted with two-colour microarrays covering 95.5% of all known *E. coli* ORFs, and the analysis method used enables a comparison to the findings from the annotation based method in this study. Courcelle *et al.* reported 163 upregulated genes following UV irradiation. Of these genes, 122 are found in the annotation used in the present study. In total 49 of the 122 genes were reported as significantly upregulated in this study and further 58 genes were reported slightly but not significantly upregulated. We present three genes, *artP*, *smpA* and *hlyE*, to be significantly downregulated and 5 genes to be insignificantly downregulated, while these genes were found to be significantly upregulated by Courcelle *et al.* [3]. The remaining seven of the 122 genes were detected with a fold change ≈0.

Comparing the 43 genes found in the downregulated operons from the Courcelle study and in the here applied annotation, 23 genes were found to be significantly downregulated, 15 insignificantly downregulated and the last five had a fold change ≈0. These minor discrepancies are most likely due to experimental biological differences combined with a rather strict definition of significant differential regulation in the present study.

Tjaden and co-workers identified operons and untranslated regions of *E. coli* using high-density oligonucleotide probe arrays [35], [36]. In the following comparisons, on genes and UTRs, only genes found in the current annotation are counted. The present study detects 176 out of the 200 known and 247 out of the 269 predicted novel operon genes detected by Tjaden *et al.* [35]. Furthermore 184 of 372 3′UTR from the Tjaden study are also found in the present study, along with 349 out of Tjaden's 528 5′UTRs. Many of the UTRs detected by Tjaden *et al.* which are not found here (163 3′UTRs and 159 5′UTRs), are detected as expressed, but presented as parts of operons in this study. Finally, a second study by Tjaden *et al.* [36] describes 340 novel transcripts as possible ORFs or ncRNAs, of which the present study detects 303 as expressed, but only 69 of these transcripts are classified as novel RNAs with no suggested function. This difference is due to the inclusion criteria applied to define transcripts as possible UTRs or as untranslated parts of operons.

### Validation of differentially expressed genes, ncRNAs and novel transcripts

**Figure 5:**
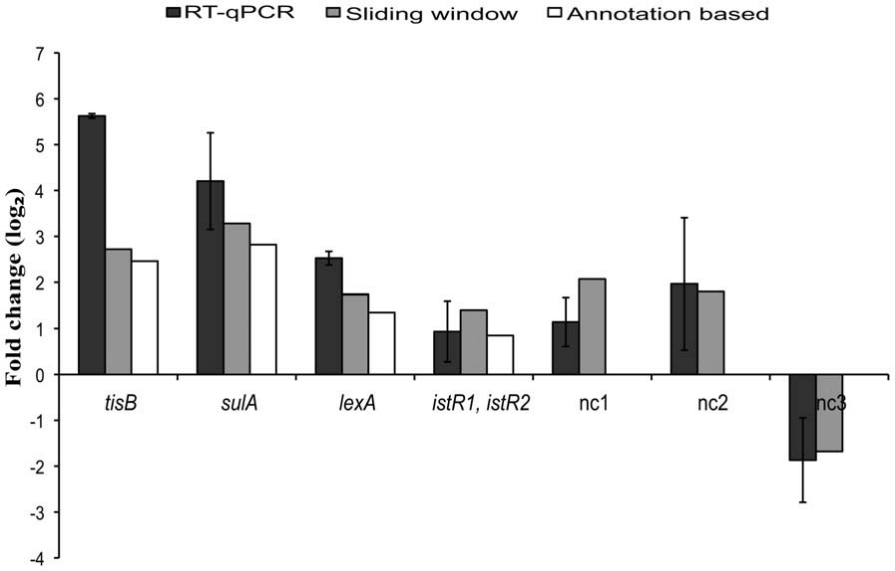
RT-qPCR validation results compared to array data. The fold change (log_2_) of the RT-qPCR validation along with the array expression data for a chosen set of differentially expressed genes and novel gene candidates. The standard errors of quadruplicate parallel RT-qPCR experiments are shown and the solid bars represent the mean of the quadruplicates. All transcript levels were measured relative to *rrsB* which was assumed to have a constant expression level. The genes examined *lexA*, *tisA/B* and *sulA* are in essence positive controls as they are well documented as being upregulated following irradiated with UV light. The fold change values assume a maximum PCR efficiency of two. The details of *nc1*-*nc3* can be found at GEO (GSE13829).

RT-qPCR was used to validate the differential expression for a selection of three genes (*tisB*, *sulA* and *lexA*), two ncRNAs (*istR-1* and *istR-2*, here detected as one continuous transcript) and three novel transcripts (nc1, nc2 and nc3) (Figure 5). The RT-qPCR was run in quadruplicates for each candidate, and all the tested transcripts were differentially expressed according to the array data. (Primer sequences are listed in Supplementary File S1). The stably expressed transcript for *rrsB* (16s rRNA) was used as the standard by which all other transcripts were measured. The stability of the *rrsB* transcript expression was confirmed by its *C_t_* value being similar between the reference and UV treated samples that contain the same quantity of RNA.

The mean expressions of the modulated intergenic transcripts were in accordance with those found by the array. The high correlation between array and RT-qPCR data along with detection of the expected upregulation of LexA sensitive genes (*tisB*, *sulA* and *lexA*) substantiate the experiment and illustrate the dramatic increases in expression levels induced by UV damage on DNA.

**Figure 6:**
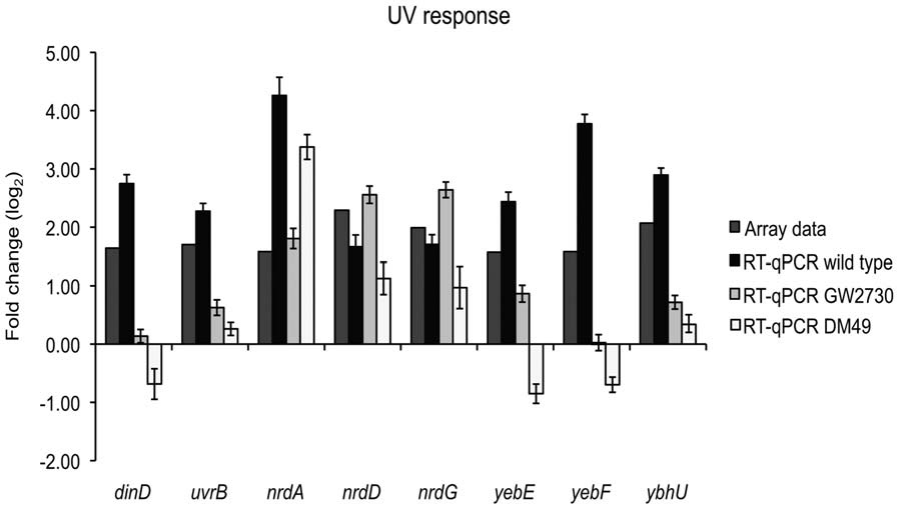
RT-qPCR of UV irradiated mutants. Fold change (log_2_) of selected genes after UV irradiation as measured by the array for MG1655 and RT-qPCR for AB1157, GW2730 (LexA(Def)) and DM49 (LexA ind-). AB1157 and MG1655 are known to have similar SOS response systems, RT-qPCR was run in triplicates and standard errors are indicated. All transcript levels were measured relative to *rrsB*, which was assumed to have a constant expression level, and the fold change values assume a maximum PCR efficiency of two.

Further we wanted to investigate the behaviour of the UV induced genes *uvrB*, *dinD*, *nrdA*, *nrdD*, *nrdG*, *yebE*, *yebF and ybhU* in two different *lexA* mutants; GW2730 *lexA71::Tn5* [LexA(Def)] and DM49 *lexA3* [lexA (ind-)] (Figure 6). As these mutants were derived from the AB1157 *E. coli* strain, we used AB1157 as reference in this particular RT-qPCR study. We confirmed the LexA dependence of *uvrB* and *dinD* along with the previously reported LexA independence of *nrdA*, *nrdD and nrdG*. The *yebE* and *yebF* genes have previously been shown as UV responsive [3], and was postulated to follow the regulation of the LexA dependent *yebG*. It seems as *yebE* is only partially LexA controlled as it shows some upregulation in GW2730, while *yebF* is under full LexA control. In the present study we detect UV induction of *yebG* as found by Courcelle *et al.* [3]. The expression pattern of *ybhU* seems almost similar to *uvrB*, hence we suggest this transcript to be LexA dependent. Further, we speculate that the predicted *uvrB* promoter sequence from within the *ybhU* ORF might be the acting cause of the LexA dependence.

### Promoter study

To investigate possible promoter sequences for up- and downregulated transcripts touching known genes we applied the online tool MEME [37] to align the 100 nt upstream of all up- and downregulated transcripts touching genes, and the 100 nt upstream of the annotated start of all differentially expressed genes. It should be noted that the p-values computed by MEME and presented in Tables 4, 5, 6, 7 and 8 are computed based on the input sequences given, and not the entire *E. coli* genome.

**Table 4:** Motif 1; upstream of induced annotated genes.

Gene	Start	P-value	Sequence(Consensus = TTTACTGTATATATATACAGTATTT)
*sbmC*	64	1.53E-12	TATACTGTATATAAAAACAGTATCA
*umuD*	58	5.47E-12	ACTACTGTATATAAAAACAGTATAA
*recN*	29	5.47E-12	TTTACTGTATATAAAACCAGTTTAT
*istR2*	37	6.63E-12	TTTACTGTATAAATAAACAGTAATA
*dinI*	59	1.91E-10	TTACCTGTATAAATAACCAGTATAT
*dinD*	34	7.87E-10	ACAACTGTATATAAATACAGTTACA
*lexA*	50	7.87E-10	TTTGCTGTATATACTCACAGCATAA
*yebG*	61	1.34E-09	TATACTGTATAAAATCACAGTTATT
*ruvA*	29	2.67E-09	TTCGCTGGATATCTATCCAGCATTT
*uvrD*	21	4.24E-09	AAATCTGTATATATACCCAGCTTTT
*sulA*	56	5.06E-09	TGTACTGTACATCCATACAGTAACT
*cho*	64	1.36E-08	TACACTGGATAGATAACCAGCATTC
*recA*	19	1.71E-08	GATACTGTATGAGCATACAGTATAA
*uvrB*	3	2.13E-08	TGAACTGTTTTTTTATCCAGTATAA
*polB*	25	9.94E-08	ATGACTGTATAAAACCACAGCCAAT
*dinG*	63	1.11E-07	AATATTGGCTGTTTATACAGTATTT
*dinB*	63	1.38E-07	ATCACTGTATACTTTACCAGTGTTG
*ftsK*	1	1.46E-07	ACTCCTGTTAATCCATACAGCAACA

The motif search was performed on the 100 nt upstream of differentially expressed genes. Start denotes the start point of the motif relative to the start of the investigated sequence.

**Table 5:** Motif 2; upstream of induced annotated genes.

Gene	Start	P-value	Sequence (Consensus = GCGCCGCTTTT)
*sdaB*	50	9.12E-07	GCGCCGCTTTC
*yhbE*	17	2.50E-06	GCGGGGCTTTT
*suhB*	58	2.50E-06	GCGCCGTTTTC
*recX*	63	3.92E-06	GCGGCCCTTTT
*pepB*	70	3.92E-06	GCGGCCCTTTT
*mutM*	37	4.57E-06	GCGGGGTTTTT
*ychM*	3	6.32E-06	GCGGGTTTTTT
*cho*	34	8.05E-06	CCGCCTCTTTT
*mnmE*	32	9.81E-06	CCGCCTTTTTT
*dinG*	8	1.19E-05	GAGCCGCTTTC
*yfdF*	27	1.85E-05	GAGCGTTTTTT
*ycfJ*	16	2.04E-05	CCGCGCTTTTC
*ryeB*	80	2.28E-05	GGGCGGTTTTT
*yeeA*	39	2.50E-05	GCGCGCCTTCT
*sulA*	13	2.71E-05	GAGGCTCTTTC
*der*	22	2.71E-05	GGGCCGTTTTC
*thiL*	63	2.71E-05	CCGGCCTTTTC
*yebE*	16	3.26E-05	GCGCATCTTTT
*hepA*	2	3.70E-05	GCGCCCTTTCC
*recF*	66	3.98E-05	GCGCGGCTTAT
*fhlA*	75	4.62E-05	GCGGTGCTTTC
*yhiN*	73	6.30E-05	GCCCGTTTTTT
*dnaA*	19	6.30E-05	CAGGGTCTTTT

The motif search was performed on the 100 nt upstream of differentially expressed genes. Start denotes the start point of the motif relative to the start of the investigated sequence.

**Table 6:** Motif 3; upstream of induced annotated genes.

Gene	Start	P-value	Sequence (Consensus = GCCTGATACGACGCTATCGCGTCGGATCGGGC)
*nrdB*	11	5.57E-18	GCCTGATAAGACGCGCCAGCGTCGCATCAGGC
*nrdG*	18	3.71E-17	GTCTGATAAGACGCGACAGCGTCGCATCAGGC
*hypF*	34	5.03E-14	GCCGGATGCGACGCTGTCGTGTCCGGCAGGGC
*hscB*	55	8.76E-14	ACCTGATTCGCCGTTATCGCGGCGGATCGCAG

The motif search was performed on the 100 nt upstream of differentially expressed genes. Start denotes the start point of the motif relative to the start of the investigated sequence.

**Table 7:** Motif 1; upstream of differentially regulated transcripts overlapping genes.

Transcript touching gene(s)	Start	P-value	Sequence (Consensus = ACTGTATATAAATACAGT)
*dinD*	37	8.90E-11	ACTGTATATAAATACAGT
*umuD, umuC*	61	3.86E-10	ACTGTATATAAAAACAGT
*recN*	32	5.04E-10	ACTGTATATAAAACCAGT
*yebG, yebF*	45	3.09E-09	ACTGTATAAAATCACAGT
*lexA, dinF*	74	3.52E-09	ACTGTATATACACCCAGG
*polB*	54	3.32E-08	ACTGTATAAAACCACAGC
*uvrD*	24	3.63E-08	TCTGTATATATACCCAGC
*uvrA*	28	7.20E-08	ACTGTATATTCATTCAGG
*recX, recA*	74	9.12E-08	ACTGTATGAGCATACAGT
*dinB*	66	9.83E-08	ACTGTATACTTTACCAGT
*uvrB*	6	1.99E-07	ACTGTTTTTTTATCCAGT
*cvpA*	30	1.17E-06	ACTGGAGCAAATCACAGC
*ybiB, ding*	66	1.31E-06	ATTGGCTGTTTATACAGT

Start denotes the start of the motif sequence counted from the start of the 100-mer upstream of the detected upregulated transcripts.

**Table 8:** Motif 2; upstream of differentially regulated transcripts overlapping genes.

Transcript touching gene(s)	Start	P-value	Sequence (Consensus = TTGACCGGCTTTTCTTTTTTTACAGGGTG)
*mnmE*	28	1.57E-12	TTGACCGCCTTTTTTCTTTTCGTAGGGCG
*yidB*	10	1.36E-10	ATCTCCGCCCTTTTTATTTCTGCAATCCG
*prfA*	59	2.09E-10	AGTACATCATTTTCTTTTTTTACAGGGTG
*thiL*	59	7.78E-09	ATCGCCGGCCTTTTCTTTTTTACCTGCTG
*add*	40	2.23E-08	TTACCCTGCTTTGTTTTTATAATGGTGCG
*ynhF*	55	4.46E-08	ATGACGGGAGATTTTTTCATCACAGTGTG
*sbmC*	0	9.41E-08	AGGCCTGACCTTTCTTTTGCAGCAGACTG
*nrdA, nrdB*	21	1.74E-07	TTCTAAGCAGCTTCCCGTACTACAGGTAG
*uvrD*	64	2.89E-07	TTCTCCGCCCAACCTATTTTTACGCGGCG
*yhdZ*	9	3.14E-07	TCTACTGGATCTTCTGTTTCAGCATGTCG
*yhdH*	19	3.40E-07	TTCATCGGCTTTGCTTTTCCATTAGCGAG
*hycC, hycB*	3	4.68E-07	AGCTGAGGCTTTGCCCGTTTTGCAGGCGT
*dinQ*	61	5.06E-07	TTGCAAGGACGTGCTGGTTTTATAACCTG
*recF*	59	5.91E-07	TGGTCGGGCGTTTCGCAGTTTGCAGATTG
*yhjH*	59	1.24E-06	AGGAACGGCGTTTTTGGTTGCAGTGTGAG
*cvpA*	66	1.54E-06	TTATCATCAGATGTTTTTTTGATTATCTG
*ryeB*	35	2.48E-06	AAAACCGCCTCAGTTCTTTCACCAGAACG

Start denotes the start of the motif sequence counted from the start of the 100-mer upstream of the detected upregulated transcript.

The MEME searches resulted in four alignments; MEME did not detect any significant motifs for any set of upstream nucleotides of the downregulated transcripts and genes. On the other hand, the search upstream of upregulated transcripts revealed several possible motifs. In the set of nucleotides upstream of the annotated start sites for all upregulated genes three different motifs were detected (Tables 4, 5 and 6). The 100 nt upstream of upregulated transcripts touching annotated genes revealed two interesting motifs (Tables 7 and 8). The top scoring motif was as expected the known SOS box consensus sequence, but the novel motifs indicate that other regulatory sequences may exist upstream of genes upregulated due to UV irradiation, as discussed below.

#### Novel small peptides

In a study by Hemm *et al.* [38] 18 novel small membrane proteins in *E. coli* K12 were presented. Their search focused on ORF searches in intergenic regions that are highly conserved between related species; additionally they searched for ORFs in regions downstream of detected ribosome binding sites (RBS). In a recent, subsequent study Hemm *et al*. [39] exhibit that many small peptides show differential expression during stress in *E. coli*. Further they argue that small proteins are an overlooked subset of stress responses in *E. coli*.

In the present study, all differentially and similarly expressed transcripts with no full or partial overlap to any annotated gene were screened for possible open reading frames (ORFs) using the GeneMark software [24]. Six differentially (Table 9) and 17 similarly expressed transcripts had putative ORFs (Supplementary File S2). None of these novel ORFs had any overlap to the 18 ORFs presented in the recent study by Hemm *et al.* [38]. Next, comparison of all differential and similarly expressed transcripts from this study to the Hemm *et al.* data show expression of 12 out of the 18 reported ORFs (Supplementary File S3). Two of these transcripts, which are partially overlapping *ythA* and *ypdK* from Hemm *et al*., show differential expression in our study. This data could indicate that *ythA* and *ypdK*, which encode putative single transmembrane peptides, may play a role in regulation of membrane associated processes important for UV protection. Protein BLAST of all 23 ORF candidates revealed that one differentially expressed and six similarly expressed ORFs had no clear homology to known proteins. Five of the differentially expressed ORFs in Table 9 (D2–D6) and twelve (S1–S7, S12–16) similarly expressed ORFs (Supplementary File S3) were similar to hypothetical proteins in other *E. coli* strains. The remaining ORFs showed similarity to proteins with suggested functions.

**Table 9:** Novel differentially expressed transcripts with ORFs.

ID	Start	End	Strand	Fold change(log_2_ scale)	Left gene	Right gene
D1	2009278	2009359	-	+1.4	*yedL*	*fliE*
D2	3634009	3634096	-	+1.4	Reverse of *yhiP* *	Reverse of *yhiP* *
D3	4529978	4530035	-	−1.4	*sgcX*	*yjhP*
D4	4533067	4533673	+	−0.8	*yjhX*	*yjhS*
D5	4570772	4571459	+	−1.4	*yjiS*	*mcrC*
D6	4571523	4571883	+	−1.17	*yjiS*	*mcrC*

*As the gene *yhiP* is not well documented the reverse strand of this gene was also included in the search space. The reverse strand sequences of well documented genes were not included due to the reverse transcription artefact problem.

**Figure 7:**
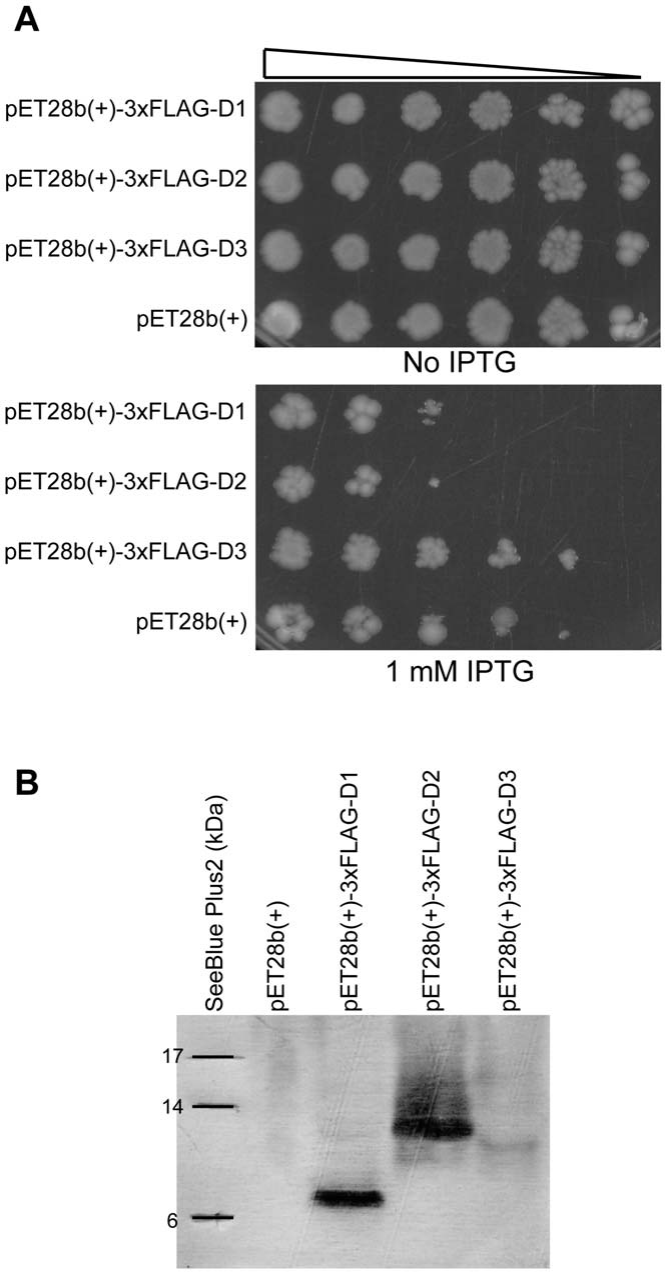
Over-expression of small novel peptides. A) To examine the toxicity of small novel peptides predicted in this work we used GenScript gene service to construct plasmids for overexpression of selected peptides. The predicted peptide sequences D1, D2 and D3 (Table 9) were cloned into the NcoI-HindIII cloning sites of the expression vector pET28b(+). Aliquots of serially diluted mid-log phase cultures of ER2566 with plasmid-constructs were spotted onto LB plates containing no IPTG (control) or 1 mM IPTG. The results demonstrated that D1 and D2 are toxic to the cell whereas D3 showed no toxicity. B) Protein expression from the pET28b(+)-D1, -D2 and -D3 plasmids were analysed by Western blotting. Total protein fractions from mid-log phase ER2566 cells transformed with expression constructs were resolved by SDS-PAGE, electroblotted to PVDF membrane and analysed using antibodies against the N-terminal FLAG tag (SIGMA).

Notably, one of the UV responsive small peptides (D1, Table 9) was predicted to form a hydrophobic single transmembrane domain, which indicates a possible role as a membrane regulatory protein. Small peptides are poorly characterized in all organisms because they are difficult to purify for biochemical characterization. Therefore, the phenotype of many chromosomally encoded small peptides and small RNA has only been reported upon overproduction from a multicopy plasmid. Many of the small peptides can be toxic to the cell, especially when overexpressed. To examine the toxicity of differentially expressed small peptides predicted in this study, we cloned the putative ORFs of D1–D3 (Table 9) containing an N-terminal 3xFLAG tag in to the expression vector pET28b(+) (See Materials and Methods for details). The constructs were transformed in to *E. coli* strain ER2566 and the transcripts were expressed under control of IPTG. The results demonstrated that D1 and D2 are toxic to the cell whereas D3 showed no toxicity (Figure 7A). Western analysis of protein extracts prepared 60 min after IPTG induction with antibodies against FLAG showed that all three peptides were expressed (Figure 7B). Although the mechanism by which elevated levels of D1 and D2 kill cells or inhibit growth is unknown, our data support that the predicted peptides are biologically active.

## Discussion

We have studied the transcriptional response to UV irradiation in *E. coli*. Many other genes than the well known SOS genes were found to be induced in *E. coli* after irradiation. Of the 170 upregulated transcripts identified, 23 potentially encode novel peptides. Three transcripts that seem to encode single transmembrane domain peptides were tested and found to be expressed; two were toxic to the cell when overexpressed. Potential regulatory sequences in addition to the SOS box upstream of the upregulated transcripts were also identified.

Both the RT-qPCR results and the detected upregulation of the majority of known UV damage repair genes bring confidence to the present findings. The number of differentially regulated genes might seem high (632 detected by annotation based method and 743 detected by the sliding window), but are nevertheless far fewer than the number reported as regulated after treatment with mitomycin C [40]. The number of upregulated genes is only slightly higher than suggested in the UV irradiation study by Courcelle *et al.* [3].

As changes induced by UV irradiation have been studied on a gene expression level before [3] the most interesting findings in this study revolve around the non-coding regions. Among the detected transcripts there are 59 ncRNA genes, and the annotation based method reports 11 differentially expressed ncRNAs while the annotation independent analysis detects 14 differentially expressed ncRNAs. The detection level of known ncRNAs is very high (94%) and when considering the concordance between the array data, which indicate a variety of novel transcripts, and the RT-qPCR verifications it is clear that the *E. coli* transcriptome houses far more transcripts than previously believed. Differential expression may indicate important functions in UV protection of *E. coli*.

In addition to the expected upregulation of the SOS genes and other known gene functions, a general downregulation as a response to UV irradiation is observed. This downregulation is in correspondence with the findings reported by Courcelle *et al.* [3] (*E. coli* MG1655 used in both studies). We therefore suggest that downregulation is a general defence mechanism that enables the bacterium to concentrate its metabolism upon genome repair. It should be noted that some of the measured downregulation might be a result of increased mRNA degradation following the exposure to UV. When examining the transcriptome data of the reference bacteria, far more transcription is seen than previous annotations suggest, which is in line with several studies published lately on other species [9], [10], [19].

The top motif of both promoter searches was, as expected, a version of the known SOS box motif [41]–[43] (Tables 4 and 7), but interestingly other consensus promoter sequences were suggested for upregulated genes as well. It has previously been shown that several LexA independent genes are regulated within a twofold range [3], with exceptions like the *nrdB* and *nrdA* genes, which show regulation beyond the twofold range [44]. Interestingly we find *nrdB* among the four genes (*nrdB*, *nrdG*, *hypF* and *hscB*) having a novel common promoter region with a remarkably low p-value (Table 6), and *nrdB* and *nrdA* are also found in Table 8 as one single transcript having a common promoter to 16 other upregulated genes, of which many are among the known SOS response genes. The four genes having the same consensus promoter cannot, according to the annotation, be put into one functional class, but it is reasonable to believe that the consensus region might be important for the regulatory mechanism. In Tables 5 and 8, genes known to have functional SOS boxes are presented and three genes (*ryeB*, *recF and mnmE*) are listed in both tables, it is also easily seen that the consensus sequence of both tables are dominated by a poly T stretch. This indicates that some of the known SOS genes might have additional binding sites for regulatory reasons. These binding sites seem to overlap with possible regulatory sequences of genes known to be LexA independent (*nrdA* and *nrdB*) and with genes detected as upregulated here, but previously undescribed as LexA independent UV damage induced genes. In summary, the large set of upregulated transcripts without a SOS box give strong indications of the existence of some alternative regulatory system that organizes these transcriptional changes. Some possible consensus regulatory sequences upstream of the transcripts have been suggested here, but further research is necessary to verify this hypothesis. The negative result of the promoter search for the downregulated transcripts is not very surprising, as a general downregulation do not require specific upstream signals.

Open reading frame search of all transcripts without overlap to any annotated gene with the GeneMark algorithm [24] revealed 23 new ORFs, in which six are differentially expressed. 17 of the open reading frames are short peptides (16–60 amino acids) and seven of these peptides show no homology to known proteins. Surprisingly, none of these ORFs were identified in a similar search by Hemm *et al*. [38] and vice versa. There are at least two reasons why we did not detect the putative peptides reported by Hemm *et al.* in our GeneMark.hmm ORF search. First, some of the Hemm *et al*. ORFs overlap with transcripts that also overlap known coding regions in our study, and consequently, they were excluded from our ORF search. Second, some transcripts in our study only has a partial overlap to the ORFs in Hemm *et al.*, resulting in missing start or stop codons in the sequences we searched with GeneMark. This observation indicates that many of the small peptides detected by Hemm *et al*. are located within transcripts overlapping known genes. The present study contains transcriptome data, which enabled us to search the expressed sequences directly for ORFs, and thus omit other filters. We believe that this difference explains why the novel ORFs presented here were missed in the Hemm *et al.* study.

Small peptides are difficult to detect and characterize using standard biochemical techniques; however, they can have diverse and universal roles in cell physiology. In a recent paper Hemm *et al.* [39] showed that many small peptides are stress induced. Two of the novel small peptides identified in this work (D1 and D2, Table 9) are upregulated, suggesting a role in UV protection. Overexpression of D1 and D2 in *E. coli* are highly toxic to the cell, indicating that both peptides are biologically active. Notably, secondary structure prediction suggests that the D1 peptide forms a hydrophobic single transmembrane domain, suggesting a possible function at the membrane. Weel-Sneve *et al*. [45] demonstrated that the SOS inducible TisB peptide, which is an inner-membrane single transmembrane domain, possesses an anti-SOS response. It therefore appears that several hydrophobic single transmembrane peptides are upregulated in response to UV damage and that these may modulate mechanisms associated with the inner-membrane, such as nucleotide excision repair, recombination repair, replication and oxidative phosphorylation [46].

### Conclusions

This work clearly demonstrates a much larger transcriptome of *E. coli* than indicated by present annotations. Several of the novel transcripts and previously undescribed UV responsive genes are differentially expressed, indicating that the *E. coli* UV response is even more complex and wide-ranging than previously shown. Further, some of these transcripts appear to have a common upstream regulatory sequence. An extensive downregulation of transcripts indicates that the bacterial stress response to UV require downregulation of numerous gene functions. Furthermore, this work confirms many of the previously *in-silico* predicted *E. coli* ncRNAs, and two UV induced peptides previously published as small inner membrane peptides [39]. Importantly, this work have predicted 23 novel small peptides and demonstrated that two of the UV induced peptides are biologically active. We conclude that the number of small peptides in *E. coli* is underestimated, which of several are important regulatory modules operating at the inner-membrane in response to UV.

## Supporting Information

File S1 **Primer sequences for RT-qPCR.**
All primer sequences used for the RT-qPCR verification
(PDF)

File S2 **The 23 novel peptides.**
In this file all NT-sequences, AA sequences, BLAST search results and the Jpred secondary structure predictions can be found for the 23 novel peptides.
(PDF)

File S3 **Overlap to previously predicted small peptides.**
All overlaps between similarly and differentially expressed transcripts from this study and the 18 small peptides predicted by Hemm *et al*. [38].
(XLS)
