# Supplementary material for: Tiling Array Analysis of UV Treated Escherichia coli Predicts Novel Differentially Expressed Small Peptides
Source: PLoS One. 2010 Dec 23;5(12):e15356. doi: 10.1371/journal.pone.0015356 (PMC3009722; doi:10.1371/journal.pone.0015356)
Supplement: File S1 — Primer sequences for RT-qPCR. All primer sequences used for the RT-qPCR verification (PDF) [file pone.0015356.s001.pdf]

Table 1 Differentially expressed genes, Mock vs UV treated cells

Gene	Forward	Reverse
lexA	AGAAGGGTTGCCGCTGGTA	TTGCGCCAGAAGTGGTTCA
tisB	ACCTGGTGGATATCGCCATTC	CTTCAGGTATTTCAGAACAGCATCAA
sulA	GCTCTCCCCTTGCCACACT	TGTAATTGCCCGTGCGTAAA

Table 2 Endogenous control gene

Gene	Forward	Reverse
rrsB	CGTGTTGTGAAATGTTGGGTTAA	CCGCTGGCAACAAAGGATAA

Table 3 Candidate differentially expressed intergenic regions & primers used, Mock vs UV treated cells

Candidate no.	Start nucleotide	Stop Nucleotide	Strand	Forward	Reverse
istR1/istR2	3851175	3851327	-	CAAAACGCAAACTGTGATCGA	CGCAATTTCGGCACGAAT
nc1	4457486	4457627	-	AATTGTCGTTGAGGGTTTCCA	TCAGTTCGGCGTCATTTGC
nc2	2342424	2342513	-	TCAGTTGTGGTCAATATATAAGCGAAT	GGCTTAGCATTTAACAATAACCGAAT
nc3	1225459	1225561	+	CCCTTGTCAATCAACCCATTG	GCGGAAAAAGGAAGCGTAAA
