# Supplementary material for: Tiling Array Analysis of UV Treated Escherichia coli Predicts Novel Differentially Expressed Small Peptides
Source: PLoS One. 2010 Dec 23;5(12):e15356. doi: 10.1371/journal.pone.0015356 (PMC3009722; doi:10.1371/journal.pone.0015356)
Supplement: File S2 — The 23 novel peptides. In this file all NT-sequences, AA sequences, BLAST search results and the Jpred secondary structure predictions can be found for the 23 novel peptides. (PDF) [file pone.0015356.s002.pdf]

Novel short ORFs detected.

Differentially expressed novel peptides

The six differentially expressed ORFs (**D1-D6**) are listed first with nucleotide sequence, amino acid sequence and JPRED structure prediction.
Nucleotide sequence includes 10 upstream bases to show possible Shine-Dalgarno sequence. Start codon is shown in **bold**. The Blast search information is described, and the top hit is listed.

Fasta header : >Position of start of ORF|Position of end of ORF|Strand|Fold change (log_2)|Regulation (up/down)

Differentially expressed small peptides:

D1
BLAST : no obvious similarities

NT seq
>2009278|2009359|-|1.43|diff_non|Upregulated
GGGATTTGCT**ATG**AAAGATCGCAGCGAAAATATATTGTTACGAATCCCGC
TGATTATGATAAATATTCCGATATGGTTAAAAAGTATGTAA

AA seq
>2009278|2009359|-|1.43|diff_non
MKDRSENILLRIPLIMINIPIWLKSM

Structure:
Query: MKDRSENILLRIPLIMINIPIWLKSM
Jpred: -----H-HHHHHHHHHHHHHHHHH--
Conf: 99986116888899999999987289

D2
BLAST : high similarity to ref|ZP_04872718.1| conserved hypothetical protein [Escherichia sp. 1_1_43]

NT seq
>3634009|3634096|-|1.41|prime3|Upregulated
GGATGGAAAG**GTG**ATTGAAAACTCACTCAGTGGCCAGATCTTTATGAAAT
ATGAACAGGTTGAAAAAAACAGTAACTTTGCTGTTTTTATACTTAA

AA seq
>3634009|3634096|-|1.41|prime3
VIENSLSGQIFMKYEQVEKNSNFAVFYT

Structure :
Query: VIENSLSGQIFMKYEQVEKNSNFAVFYT
Jpred: ---------EEEEEEE------EEEEE-
Conf: 6888888607877760478873688736

D3
BLAST : high similarity in the N-terminal end to ref|ZP_06167183.1| glyoxalase I, nickel isomerase [Klebsiella variicola At-22]

NT seq
>4529978|4530035|-|1.4|diff_non|Downregulated
AGGAGATCCG**ATG**TTTTCTTACATTATGCTGGGAACCAACGACCTGCCAC
GCGCCATTACATTCTAA

AA seq
>4529978|4530035|-|1.4|diff_non
MFSYIMLGTNDLPRAITF

No structure predicted - too short

D4
BLAST : high similarity to ref|YP_002394396.1| putative frameshift suppressor; KpLE2 phage-like element [Escherichia coli S88]

NT seq
>4533067|4533673|+|0.8|diff_non|Downregulated
TGGTAGAGGC**ATG**AAGCCAAATGGAGGGAGCCGTCATAACCCTCTTGAA
GCTGAAACAATAGCAGCATGGCTGGTGGCACATAAGGATGATATAGAGC
GCCATTACGGGGAACCACTGTACAAAGTTGTTGGTGTTGTGACGCCATTTT
CGGCGCAGGTTAATGCTATCAAAATGTCATTGCGTAAGCTGGAAATTAAT
GGCAAAGACGAGCAGGGTTTACTGACAGTGGGGACTGTGCACTCCTTGCA
GGGGGCGGAAAGGGCGATTGTTCTCTTTTCTCCTGTCTACTCAAAACATGA
AGATGGCAGGTTTCTTGATAGCAACAGCACTATCCTCAATGTTGCTGTCTC
ACGCGCTAAAGATAGTTTTCTGGTATTTGGCGATATGGACCTTATCGAGAT
GCAGCCCGCATTTTCGCCGCGAGGGTTATTGGCAAAATATCTCTTTTCTTC
AGATAATAATGCGTTGCAATTTGAGTTTCAGAAACGACAGGATTTAATTT
CCGCACATACACAAATTTCAACGCTGCATGGTGTGGAGCAGCATGATGAA
TTTTTGAACAAGACTCTTGCCGGAGCTCAAAAGAAAATAACGATTATTTC
CCCCGAGATATAG

AA seq
>4533067|4533673|+|0.8|diff_non
MKPNGGSRHNPLEAETIAAWLVAHKDDIERHYGEPLYKVVGVVTPFSAQVN
AIKMSLRKLEINGKDEQGLLTVGTVHSLQGAERAIVLFSPVYSKHEDGRFLDS
NSTILNVAVSRAKDSFLVFGDMDLIEMQPAFSPRGLLAKYLFSSDNNALQFEF
QKRQDLISAHTQISTLHGVEQHDEFLNKTLAGAQKKITIISPEI

Structure:
Query:
MKPNGGSRHNPLEAETIAAWLVAHKDDIERHYGEPLYKVVGVVTPFSAQVN
AIKMSLRKL

Jpred: -------EE-HHHHHHHHHHHHHHHHHHHHHH--------EEEEE--
HHHHHHHHHHHHHH
Conf: 99988612035899999999999999998702688888457873565889999999870

Query:
EINGKDEQGLLTVGTVHSLQGAERAIVLFSPVYSKHEDGRFLDSNSTILNVAV
SRAKDSF
Jpred: ----------EEEEE---------EEEEEEEE---------------HHHHHHHHH---E
Conf: 26777788745887004677777048898740677777751157600788886764000З

Query:
LVFGDMDLIEMQPAFSPRGLLAKYLFSSDNNALQFEFQKRQDLISAHTQISTL
HGVEQHD
Jpred: EEEE--HHH-------HHHHHHHHHHHH-----E-----------------H-----HHH
Conf: 67743023225788740899999988507884000267777777765200002550009

Query: EFLNKTLAGAQKKITIISPEI
Jpred: HHHHHHH----EEEEEE----
Conf: 999987548964889861799

D5
BLAST : high similarity to ref|AP_004833.1| hypothetical protein [Escherichia coli str. K-12 substr. W3110]

NT seq
>4570772|4571459|+|1.4|diff_non|Downregulated
ACCGAAAGGG**ATG**GAAGATTACTGGCTGCGCCCAATTCGATTTTATGAAT
CTGAACGACGCAATTTTCTCGGAACATTATTCAGTGAAGGTGGTTTACCTT
TCCGGCTACTCAAAGAATCAGACAGTCGCTTTCTGGCTGTTTTCTCCAGGA
TCCTTGGACAATATGAGCAAGCAAAGCAGTCAGGTTTTTCTGCCCTTTCGC
TGGCAAGAGCTGTCATAGAAAAATCAGCGCTGCCAACTGTTTTTAGCGAA
GATACCTCTGTAGAACTTATCAGTCATATGGCTGATAACCTCAATTCCTTA
GTACTTACTCACAACCTAATAAATCACAAAGAACCAGTACAACAGCTTGA
GAAGGTTCACCCTACCTGGCGAAGCGAGTTTCCAATACCGTTAGATGATG
AAACAGGAACGCATTTTTTGAATGGACTGCTATGCGCAGCCTCTGTAGAG
GCTAAGCCTCGACTACAAAAAAATAAGAGTACGCGTTGCCAATTCTATTG
GTCAGAAAAGCATCCAGATGAGCTTAGGGTGATAGTATCTCTTCCGGACG
AAGTTTCCTTTCCTGTAACAAGCGAGCCGTCAACTACGCGCTTTGAACTTG
CCATTTGTGAAGATGGTGAGGAAGTCTCTGGCCTTGATCTATACCGGTGG
CGATACGATTACACGAAGCGGAGAAACTGTAACGCTAAATAA

AA seq
>4570772|4571459|+|1.4|diff_non
MEDYWLRPIRFYESERRNFLGTLFSEGGLPFRLLKESDSRFLAVFSRILGQYEQ
AKQSGFSALSLARAVIEKSALPTVFSEDTSVELISHMADNLNSLVLTHNLINHK
EPVQQLEKVHPTWRSEFPIPLDDETGTHFLNGLLCAASVEAKPRLQKNKSTRC
QFYWSEKHPDELRVIVSLPDEVSFPVTSEPSTTRFELAICEDGEEVSGLDLYRW
RYDYTKRRNCNAK

Structure :

Query:
MEDYWLRPIRFYESERRNFLGTLFSEGGLPFRLLKESDSRFLAVFSRILGQYEQ
AKQSGF
Jpred: --------EEEE------EEEEEEE-----HHHH------HHHHHHHHHHHHHHHH-----
Conf: 99855750006248887368886405776500000478755789999999862068887

Query:
SALSLARAVIEKSALPTVFSEDTSVELISHMADNLNSLVLTHNLINHKEPVQQL
EKVHPT
Jpred: HHHHHHHHHHH---HHHHH----HHHHHHHHHHHHHHHHHHHHH-------
HHHHHHHH---
Conf: 068899988500750120077746899999999999999860578875078898874266

Query:
WRSEFPIPLDDETGTHFLNGLLCAASVEAKPRLQKNKSTRCQFYWSEKHPDE
LRVIVSLP
Jpred: -----------HHHHHHHHHHHHHHHHHHHH--------EEEEEEEE----
EEEEEEEEE-
Conf: 655677787651799999999999998750678877725889987167604789999844

Query:
DEVSFPVTSEPSTTRFELAICEDGEEVSGLDLYRWRYDYTKRRNCNAK
Jpred: -EEEEE---------EEEEEEE----------HHH------------
Conf: 308880268877750578775046005776100000500544567889

D6
BLAST : high similarity to ref|YP_002415529.1| hypothetical protein ECUMN_4952 [Escherichia coli UMN026]

NT seq
>4571523|4571883|+|1.17|diff_non|Downregulated
GCTGGAGGG**ATG**ATTGTTGGGAGCATTAAACTTGATGACAGTGAAATTGC
CATTGGTGAGGTGCCGTTAACCTTCATCGTTGATGCAGATCAATGGCTGTT
ACAGGGACAGGCTTCTTGCAGTGTGCGAAGCAGCGATGTTCTGATTGTGC
TCCCTCGGGATAATAGCAATGTTGCTGGTTTTGATGGCCAATCGAGGGCA
GTAAACGTATTAGGACTAAAAGCACTACCTGTGAAGGGATGTCAGGACGT
TACAGTTACAGCAAATGAAACTTATCGGATTCGCACCGGAAGAGAACAAA
TCAGCATCGGAAGGTTTGCTCTAAATGGAAAGCGTGCAAGCTGGGTTTGT
CATCCAGATGAAACATAG

AA seq
>4571523|4571883|+|1.17|diff_non
MIVGSIKLDDSEIAIGEVPLTFIVDADQWLLQGQASCSVRSSDVLIVLPRDNSN
VAGFDG
QSRAVNVLGLKALPVKGCQDVTVTANETYRIRTGREQISIGRFALNGKRASW
VCHPDET

Structure:

Query:
MIVGSIKLDDSEIAIGEVPLTFIVDADQWLLQGQASCSVRSSDVLIVLPRDNSN
VAGFDG
Jpred: -EEEE--------------EEEEEE--EEEEEE-----E---EEEEEE------------
Conf: 62652004776545776200898507515677347750237606888536777777777777

Query:
QSRAVNVLGLKALPVKGCQDVTVTANETYRIRTGREQISIGRFALNGKRASW
VCHPDET
Jpred: ----EEEE------------EEE----EEEEEE-----EE--EEE--EEEEEE------
Conf: 76650000256770077762577068558998547850500077705046656037899

Similarly expressed small peptides

The 17 differentially similarly expressed ORFs (S1-S17)are listed with nucleotide sequence, amino acid sequence and JPRED structure prediction. The nucleotide sequence includes 10 nucleotides upstream of the start codon to show possible Shine-Dalgarno sequence, the start codon is in **bold.**
Fasta header: > Position of start of ORF|end position of ORF|strand

S1
BLAST : high similarity to nucleotide sugar synthetase [Escherichia coli W3110]

NT seq
>2101098|2101158|-
TGATTACATT**TTG**TTTTTGAATCCCGATATCATCATGAAGCATGATGATTT
GCTGACATATATTAAATAT

AA seq
>2101098|2101158|-
LFLNPDIIMKHDDLLTYIKY

Structure :
Query: LFLNPDIIMKHDDLLTYIKY
Jpred: ------EEE----HHHH---
Conf: 87467007703642322079

S2
BLAST high similarity to gb|AAB40749.1|outer membrane porin protein [Escherichia coli]

NT seq
>575577|576048|-
AGGGCTCACG**ATG**AAAAAATTAACAGTGGCAATTTCTGCTGTAGCTGCAT
CAGTACTAATGGCGATGTCTGCTCAGGCAGCTGAAATTTATAATAAAGAC
AGTAACAAGCTGGATCTGTACGGGAAAGTTAATGCTAAGCACTACTTCTC
CTCTAATGATGCAGATGATGGTGATACTACTTATGCCCGTCTTGGCTTCAA
AGGTGAAACCCAAATCAACGATCAACTGACTGGTTTCGGTCAGTGGGAAT
ATGAATTCAAAGGCAACCGCGCTGAATCTCAAGGTTCCTCCAAAGATAAA
ACCCGTCTTGCCTTCGCTGGCCTGAAATTCGGTGACTACGGCTCCATCGAT
TACGGCCGTAACTACGGTGTAGCATACGACATCGGTGCGTGGACTGACGT
CCTGCCAGAATTCGGTGGTGACACTTGGACTCAAACCGACGTGTTCATGA
CTCAACGTGCAACTGGTGTTGCAACCTAT

AA seq
>575577|576048|-
MKKLTVAISAVAASVLMAMSAQAAEIYNKDSNKLDLYGKVNAKHYFSSND
ADDGDTTYARLGFKGETQINDQLTGFGQWEYEFKGNRAESQGSSKDKTRLA
FAGLKFGDYGSIDYGRNYGVAYDIGAWTDVLPEFGGDTWTQTDVFMTQRAT
GVATY

Structure :

Query:
MKKLTVAISAVAASVLMAMSAQAAEIYNKDSNKLDLYGKVNAKHYFSSND
ADDGDTTYAR
Jpred: ---HHHHHHHHHHHHHHHHHHHHHHHEE-----EEEEEEEEEEEEEEE--------
---EE
Conf: 87338999999999997555441000548884588988888899986146777777774009

Query:
LGFKGETQINDQLTGFGQWEYEFKGNRAESQGSSKDKTRLAFAGLKFGDYGS
IDYGRNYG
Jpred: EEEEEEEE-----EEEEEEEEEEE--------------EEEEEEE----EEEEEEE----
Conf: 888768870675089988889986047777777777710889985488617888715886

Query: VAYDIGAWTDVLPEFGGDTWTQTDVFMTQRATGVATY
Jpred: HHHHHH--------------------------EEE--
Conf: 0000000577777777777777777777410103327

S3
BLAST : high similarity to ref|NP_313240.2| Gene info cytochrome b(562)
[Escherichia coli O157:H7 str. Sakai]

NT seq
>4457575|4457872|+
CGAAGACAAT**ATG**GAAACCCTCAACGACAATTTAAAAGTGGTCGAAAAA
GCCGATAACGCGGCGCAAGTCAAAGACGCGTTAACGAAGATGCGCGCCG
CAGCGCTGGATGCGCAAAAAGCAACGCCGCCGAAGCTCGAAGATAAATC
ACCGGACAGCCCGGAAATGAAAGATTTCCGCCACGGTTTCGACATTCTGG
TCGGTCAGATTGACGACGCGCTGAAGCTGGCAAATGAAGGTAAAGTAAA
AGAAGCGCAGGCTGCTGCAGAGCAACTGAAAACGACCCGCAATTCATATC
ACAAGAAGTAT

AA seq
>4457575|4457872|+
METLNDNLKVVEKADNAAQVKDALTKMRAAALDAQKATPPKLEDKSPDSP
EMKDFRHGFDILVGQIDDALKLANEGKVKEAQAAAEQLKTTRNSYHKKY

Structure :
Query:
METLNDNLKVVEKADNAAQVKDALTKMRAAALDAQKATPPKLEDKSPDSP
EMKDFRHGFD
Jpred: -HHHHHHHHHH-----HHHHHHHHHHHHHHHHHHHHH-------------
HHHHHHHHHH
Conf: 806999999860788668999999999999999861467777777777006899999999

Query: ILVGQIDDALKLANEGKVKEAQAAAEQLKTTRNSYHKKY
Jpred: HHHHHHHHHHHHHHH---HHHHHHHHHHHHHHHHHHHH---

Conf: 9999999999998705658999999999999999860589

S4
BLAST: no obvious similarities

NT seq
>3358750|3358834|-
TAACAAGTAA**TTG**AGACGGATTGTAAATGAGAAATTGCTTGATGTGCTTC
ACTCATCAGACTTGGTGAACAAAGCACAATTTATTGAAAATTAT

AA seq
>3358750|3358834|-
LRRIVNEKLLDVLHSSDLVNKAQFIENY

Structure :
Query: LRRIVNEKLLDVLHSSDLVNKAQFIENY
Jpred: --HHHHHHHHHHH-----HHHHHHHH--
Conf: 9848999999875378855774776279

S5
BLAST : high similarity to malate dehydrogenase [Escherichia coli UTI89]

NT seq
>3382305|3382356|-
AACGGCAATT**TTG**TGGATTAAGGTCGCGGCAGCGGAGCAACATATCTTAG
TTTATCAATAT

AA seq
>3382305|3382356|-
LWIKVAAAEQHILVYQY

Structure : Too short to predict

S6
BLAST : high similarity to hypothetical protein EcE24377A_0429 [Escherichia coli E24377A]

NT seq
>418433|418529|-
TGAAAAAGAC**ATG**GCTAATCATGCGAACAAAAAATCACCAATCGTTGTA
AAATCTAACGCCCGTATTTTGACTGCAAAGAAAATAAGCCAGCATCGACT
GGCTTAT

AA seq
>418433|418529|-
MANHANKKSPIVVKSNARILTAKKISQHRLAY

Structure :

Query: MANHANKKSPIVVKSNARILTAKKISQHRLAY
Jpred: ----------EEEE----EEEE-HH-H-----
Conf: 99888877736885478457612330057999

S7
BLAST : exactly similar to: hypothetical protein EcF11_2204 [Escherichia coli F11].

NT seq
>271775|271883|+
GTGTTTTGAC**ATG**TATGCGAAACACCGGGGCAGCAGCGTCTATCACGCTT
GCGTTGCTGACTTCTGCCAACTTGCGGCAAGCAAGGATAAAGAGTGCGAC
GGGCAGCCTCCTCAGTAT

AA seq
>271775|271883|+
MYAKHRGSSVYHACVADFCQLAASKDKECDGQPPQY

Structure :
Query: MYAKHRGSSVYHACVADFCQLAASKDKECDGQPPQY
Jpred: ----------HHHHHHHHHHHHH-------------
Conf: 986677775008999999998702677777777888

S8
BLAST : no obvious similarities

NT seq
>1014684|1014786|-
GGCATGTCGG**TTG**TACAAAAAATGTGAAGAGGAACAAATTTTTCCGCACG
TTAGAGACAAAAAAACCGCCTCAGGCAACATACCGGAGACGGTTATATTT
ACAAATCTTTAT

AA seq
>1014684|1014786|-
LYKKCEEEQIFPHVRDKKTASGNIPETVIFTNLY

Structure :
Query: LYKKCEEEQIFPHVRDKKTASGNIPETVIFTNLY
Jpred: -------------------------EEEE----
Conf: 9988877777777777777777515635899

S9
BLAST : no obvious similarities

NT seq
>1801151|1801202|-
ATAGGCTCTG**ATG**AAGGTGTTGGTATGTCGCTATCGATGTGCAATCTATG
AGGAGACTTAT

AA
>1801151|1801202|-

MKVLVCRYRCAIYEETY

Structure : Too short to predict

S10
BLAST : no obvious similarities

NT seq
>1677411|1677483|-
TTACGAAGAG**ATG**TGTCTGCATGCAATGGTCGCGGGGAAATTTGTGACTA
AAAATGCGAAAGGCGGCATCAAGCCGCCTTAT

AA seq
>1677411|1677483|-
MCLHAMVAGKFVTKNAKGGIKPPY

Structure :
Query: MCLHAMVAGKFVTKNAKGGIKPPY
Jpred: ---HHHHH--EEE-----------
Conf: 997466534536437777778899

S11
BLAST : no obvious similarities

NT seq
>3672497|3672548|-
TCCCAAATAG**ATG**GCTGAAGAGGCTCTGGGGACGAGTTTCAAGAGGTTAT
GTGCGCATTAT

AA seq
>3672497|3672548|-
MAEEALGTSFKRLCAHY

Structure: too short to predict

S12
BLAST : no obvious similarities

NT seq
>892871|892937|-
TGCAATAAAC**GTG**CATATTTTTATCGGCGCGGTGTAAGAAAAGAGAACT
TTCTCCCGGCCAGCACTGCTCGCTAT

AA seq
>892871|892937|-
VHIFYRRGVRKENFLPASTARY

Structure :

Query: VHIFYRRGVRKENFLPASTARY
Jpred: ---H------------------
Conf: 9710146777777777778899

S13
BLAST : exactly similar to hypothetical protein ECO26_4577 [Escherichia coli O26:H11 str. 11368]

NT seq
>3632079|3632250|-
TAGTCACCAG**ATG**GAGAAAGTATGGTTTATAATAGTGAACCTTCTATAG
ATATTCAAGAATATAATAGATTCGCCGACAATACTACTGATACGTTTATCT
TTACAATTATACCAGACAATAACCATGTGTTAAAATTATCGTCTCCCATTA
CCGTTACTGTTGAGTGTAAGGGCGGTTAT

AA seq
>3632079|3632250|-
MEKVWFYNSEPSIDIQEYNRFADNTTDTFIFTIIPDNNHVLKLSSPITVTVECKG
GY

Structure :
Query:
MEKVWFYNSEPSIDIQEYNRFADNTTDTFIFTIIPDNNHVLKLSSPITVTVECKG
GY
Jpred: --EEEE---------HH----------EEEEEEE----EEEEE----EEEEEE----
Conf: 980440688765222000567777787058898548862788548508999844899

S14
BLAST : similar to hypothetical protein CKO_01089 [Citrobacter koseri ATCC BAA-895].

NT seq
>1956449|1956515|-
GTCGCCCAAT**ATG**ATGCTTGCTCGTACCAGGCCCCTGCAATTTCAACAGG
GGCCTTTTTTATCCCTGAACAGTAT

AA seq
>1956449|1956515|-
MMLARTRPLQFQQGPFFIPEQY

Structure :
Query: MMLARTRPLQFQQGPFFIPEQY
Jpred: ----------------------
Conf: 9988877777777764257899

S15
BLAST : exactly similar to hypothetical protein ECIAI1_2752 [Escherichia coli IAI1].

NT seq

>2783625|2783718|-
TCTATATTTC**ATG**ACTAACGCCATTTCAAGGAAGAAGTGCGGTGGGTGGT
TTCGAATGAGGGCTGGTTCTATATTTCGTTTTGGTACGTGGCACGCCACTG
CAATGTATTTAT

AA seq
>2783625|2783718|-
MTNAISRKKCGGWFRMRAGVLVRGTPLQCIY

Structure :
Query: MTNAISRKKCGGWFRMRAGVLVRGTPLQCIY
Jpred: ---HHHHHH----EEE--EEEEE--------
Conf: 9855777504670450010898036544126

S16
BLAST : exactly similar to hypothetical protein ECIAI1_2985 [Escherichia coli IAI1]

NT seq
>2997966|2998023|-
CCTTTGCCAA**ATG**AAATTAACCTCTTTCTCTATTACCACGTTTTCCAGAA
GCAAGAGATTGGGTAT

AA seq
>2997966|2998023|-
MKLTSFSITTFFQKQEIGY

Structure : Too short to predict

S 17
BLAST : exactly similar to hypothetical protein E2348C_1940 [Escherichia coli O127:H6 str. E2348/69].

NT seq
>1899629|1899806|-
CGTCTCTGAT**ATG**TTTAACTCCCGTTTAACAACCATGGAGTATAGGGCAGT
AGCCCGCAGTATGGATCGTCACCGACGTCATTTCAGCATCAGGCCTTTTAA
CGCCTGCCTTTCTGGCACTCTATGCCGCACCTTTCGTTTGCATTTTGTCGTT
ACGCCTGCATTATTTCTGGCGTCGAATAGCTAT

AA seq
>1899629|1899806|-
MFNSRLTTMEYRAVARSMDRHRRHFSIRPFNACLSGTLCRTFRLHFVVTPALF
LASNSY

Structure :
Query:MFNSRLTTMEYRAVARSMDRHRRHFSIRPFNACLSGTLCRTFRLHFVV
TPALFLASNSY
Jpred: ---EEEEEHHHHHHHHHHH------------H-HHH-HH---EEEEEEE--HHH------
Conf: 85000000026889900000000232222000000000345178987320011057899
